# MicroRNAs in Human Malignant Gliomas

**DOI:** 10.1155/2012/732874

**Published:** 2012-07-10

**Authors:** Masahiro Mizoguchi, Yanlei Guan, Koji Yoshimoto, Nobuhiro Hata, Toshiyuki Amano, Akira Nakamizo, Tomio Sasaki

**Affiliations:** Department of Neurosurgery, Graduate School of Medical Sciences, Kyushu University, 3-1-1 Maidashi, Higashi-ku, Fukuoka 812-8582, Japan

## Abstract

MicroRNA (miRNA) is a new class of small noncoding RNA molecules that regulate a wide spectrum of gene expression in a posttranscriptional manner. MiRNAs play crucial roles in tumorigenesis, angiogenesis, invasion, and apoptosis for various types of tumor. Recent studies have identified dysregulation of specific miRNAs in malignant gliomas. Global expression profiling of miRNAs has revealed several miRNAs clinically implicated in human glioblastomas. Some miRNAs are clearly associated with clinical outcome and chemo- and radio-therapy resistance in these tumors. Furthermore, miRNAs also regulate specific signaling pathways, including the critical core pathways in glioblastoma. As a result, miRNAs have the potential to affect the responses to molecular-targeted therapies. More recent studies have revealed that miRNAs might be associated with cancer stem cell properties, affecting tumor maintenance and progression. Recent investigation have revealed that miRNAs are not only biological markers with diagnostic implications, but also one of the most promising treatment targets in human glioblastoma. Herein, we summarized the novel insights of miRNAs into human malignant gliomas.

## 1. Introduction

Glioblastoma is one of the most common and malignant primary brain tumors in adults. Glioblastoma is essentially a pathogenetically heterogeneous tumor, and resent large-scale genomic analyses have allowed molecular subclassification of glioblastomas [1–3].

 Recent work has identified a class of small noncoding RNA molecules, named microRNA (miRNA), which regulate a wide spectrum of gene expression in a posttranscriptional manner [4, 5]. More than 1500 precursors and 1921 mature human miRNAs have been discovered and registered in miRBase to date [6], and these miRNAs reportedly regulate approximately 30% of all protein-coding genes [7]. At present, it is predicted that more than half of protein coding genes would be regulated by miRNAs. The incorporation of the mature miRNA into an effector complex, called RNA-induced silencing complex (RISC), binds to messenger RNA (mRNA) and can affect the translation and stability of mRNA [7]. Recent reports have revealed that miRNAs play crucial roles in tumorigenesis, angiogenesis, invasion, and apoptosis in various types of tumor [4, 5]. In addition, previous miRNA expression profiling may yield more accurate classification of human cancers than mRNA expression profiling [8]. A recent large-scale multidimensional analysis of molecular characteristics, The Cancer Genome Atlas (TCGA), which includes the expression profiles of miRNA as well as DNA copy number, gene expression, and DNA methylation, has revealed frequent genetic alterations in three critical core pathways [9]. The present paper summarizes recent insights regarding miRNA and human malignant gliomas.

## 2. Identification of MicroRNAs

 In 2002, Calin first reported the frequent deletions and downregulation of miR-15 and miR-16 at chromosome 13q14 in a majority of cases of chronic lymphocytic leukemia (CLL) [10] and also demonstrated that more than half of miRNA genes are located in cancer-associated genomic regions or in fragile sites [11]. In addition, Lu et al. reported that the classification of malignant tumors based on miRNA expression profiles was more precise than mRNA profiles [8]. These findings have facilitated miRNA research in the field of oncology. In 2005, two groups initially reported miRNA dysregulation in glioblastoma using microarray analysis [12, 13]. Chan et al. identified five upregulated and three downregulated miRNAs out of 180 miRNAs in glioblastoma, while Ciafré et al. also demonstrated nine upregulated and four downregulated miRNAs. A common miRNA identified by both groups was miR-21, which was revealed to act as an antiapoptotic factor [12, 14]. Both groups used microarray analysis to identify miRNAs, whose expression was altered in glioblastoma. Subsequently several studies by using microarray analysis have also reported. Godlesski et al. and Sasayama et al. reported additional miRNAs dysregulated in glioblastoma [15, 16]. They also investigated the biological function of miR-128 and miR-10b, respectively [15, 16]. We subsequently applied the stem-loop reverse transcription-PCR array, which can evaluate miRNA expression with sensitivity and specificity superior to microarrays [17, 18]. Our study identified 16 miRNAs for which expression was significantly altered in glioblastoma (WHO grade 4) compared with anaplastic astrocytoma (WHO grade 3). Furthermore, we clearly demonstrated that overexpression of miR-196a or -196b is correlated with shorter overall survival among patients with malignant glioma [19]. In the same year, Malzkorn et al. also identified 12 upregulated miRNAs involved in the malignant progression of gliomas using stem-loop real-time RT-PCR [20]. Kim et al. also reported clinical implications of miR-26 gene amplification in glioblastoma patients by using TCGA data [21]. Recently, Rao et al. performed locked nucleic acid (LNA) array for a large-scale, genomewide miRNA expression profile and identified 55 upregulated and 29 downregulated miRNAs in malignant gliomas [22]. More important thing of this study was that a cluster of only 23 miRNAs was sufficient to distinguish glioblastoma from anaplastic astrocytoma with an accuracy of 95% [22]. Furthermore, Srinivasan et al. identified a ten-miRNA expression signature, which could predict overall survival of glioblastoma patients [23]. More recently, combination of mRNA and miRNA expression profiling signature identified five glioblastoma subclasses [24].

 In this paper, miRNAs dysregulated, which were identified by recent global studies for glioblastomas, were summarized (Table 1). Each report has demonstrated several miRNAs, whose expression was significantly altered in glioblastoma compared with normal brain tissues or lower-grade gliomas. Taken together, 52 upregulated miRNAs and 33 downregulated miRNAs have been reported by seven global studies published between 2005–2010 (Table 1). Interestingly, the results of each report were quite varied. One of the causes of this diversity is the difference of methodology, platform, and control samples. Consequently novel miRNAs, of which more than three studies confirmed aberrations, were only four miRNAs (miR-21, miR-10b, miR-128-1, and miR-128-2). In fact, newly identified miRNAs have not been elucidated in previous analysis; however, the number of concordance among these studies is extremely small. It would be better to say that these four miRNAs may be consistently important in glioblastomas.

## 3. miR-21

One of the most common miRNAs identified by recent studies of gliomas, miR-21 has been revealed to act as an anti-apoptotic factor that targets a network of p53, transforming growth factor (TGF)-*β*, and mitochondrial apoptosis tumor suppressor genes in glioblastoma cells [12, 14]. Overexpressed miR-21 is a unique miRNA, whose overexpression is identified in a large number of cancers investigated to date. Essentially, Medina et al. first demonstrated that tumors addicted to oncomiRs by using an *in vivo* model of miR-21-induced pre-B-cell lymphoma [25]. This result has facilitated the research elucidating the potential application as therapeutic targets in cancer. Our data revealed that miR-21 expression is significantly higher in glioblastoma than anaplastic astrocytoma (3.6 fold); however, the expression did not affect patients' outcome [19]. The important point to note is that miR-21 is only one miRNA, whose overexpression is identified in all of the recent reports. In addition, previous large study identified miR-21 upregulation in all of tumor types [26]. MiR-21 is considered to be one of the critical miRNA in tumorigenesis.

## 4. miR-196

We have already reported a significant association between high expression of miR-196 and shorter overall survival among glioblastoma patients [19]. Expression of miR-196a and miR-196b is extremely high compared with other overexpressed miRNAs in glioblastoma, so both of these two miRNAs are considered to be associated with the malignant transformation of gliomas [19]. Overexpression of miR-196 has also recently been identified in several cancers [27]. Bloomston et al. first reported the clinical implications of miR-196a-2 in patients with pancreatic cancer [28]. Our data also showed correlations between overexpression of miR-196 and shorter overall survival with malignant gliomas [19]. The characteristic of miR-196 is the location in the vicinity of homeobox (HOX) clusters within the genome of vertebrate genomes [29]. Three miR-196 genes have been identified; miR-196a-1 located on chromosome 17 (17q21.32) at HOXB cluster, miR-196a-2 located on chromosome12 (12q13.13) at HOXC cluster, and miR-196b is located on chromosome 7 (7p15.2) at HOXA cluster. Interestingly, another miRNA involved in the HOX cluster, miR-10b, appears strongly upregulated in glioma. Recent reports have also indicated clinical implications of HOX genes in glioblastoma [30, 31], so the implications of miRNA dysregulations in HOX clusters should be clarified for glioblastomas. The function of miR-196 is, however, more complicated, since the targets of miRNA usually number more than one hundred. Another target of miR196a, Annexin A1 (ANXA1), has also been implicated in glioblastoma as a mediator of apoptosis and an inhibitor of cell proliferation [32]. Previous study, which evaluated miRNA expression in human cancer cell lines, also showed overexpression of miR-196 in glioblastoma cell lines [27]. However, Lakomy et al. reported an association opposite to our finding between miR-196b expression and clinical outcome, indicating another important aspects [33]. Further investigation is necessary to elucidate the clinical implications and biological functions of miR-196 in malignant gliomas.

## 5. miR-10b

 Upregulation of miR-10b is one of the common aberrations in glioblastoma, which is identified by several recent reports [13, 15, 16, 24, 34] (Table 1). Importantly, miR-10b is overexpressed in the vast majority of glioblastoma, whereas it is not detected in normal brain. Previous reports showed that miR-10b was overexpressed in breast cancer and regulated invasion and metastasis by targeting HOXD10 [35, 36]. Sasayama et al. reported that the expression of RhoC and uPAR was correlated with miR-10b expression level and lead to multifocal and dissemination of glioblastoma [16]. They indicated that miR-10b was associated with the invasion and migration of glioma cells [16]. However, Gabriely et al. demonstrated that in glioblastoma miR-10b regulated cell cycle and programmed cell death via regulation of Bim, TFAP2C, p16, and p21 but not HOXD10 and was significantly associated with patients' survival [34]. In glioblastoma, the function of miR-10b appeared to be different from breast cancer. However, it remains under investigation whether cellular environments affect the function of miR-10b.

## 6. miR-128

Godlewski et al. identified downregulation of miR-128 in glioblastoma compared with adjacent brain, leading to a reduction in self-renewal of glioma stem cells via Bmi-1 downregulation [15]. That report was the first demonstration of an association between miRNA and stem-cell properties in glioma. In addition, TCGA revealed that miR-128 expression is lower in high-grade gliomas than in low-grade gliomas. A recent study revealed that reduced miR-128 levels are associated with dedifferentiation and aggressiveness of malignant gliomas via EGFR/PDGF/AKT signaling [37]. Interestingly, miR-128 is enriched in brain, and associated with terminally differentiated neuron [38, 39]. Verhaak et al. have revealed that miR-128 was repressed in glioblastoma classified the aggressive/dedifferentiated tumor subtype [2]. MiR-128 has been shown to be able to repress the growth of glioma initiating cell *in vivo* [37]. These findings support that miR-128 has a potential to achieve its therapeutic effect by suppressing proliferation and enhancing differentiation of glioma initiating cells.

## 7. Subclassification Based on miRNA ****Expression Profiles

Two classifications based on mRNA expression profiles have been reported in recent year, and both identified one of the subclasses as showing an expression signature resembling that of a “proneural” precursor cell [1, 2]. However, the other subclasses of glioblastomas have shown little association with neural differentiation, and disagreement remains regarding the relationship of the subclasses to clinical outcomes [1, 2]. Establishment of more accurate diagnostic and therapeutic strategies based on pathogenetic subclassification is obviously clinically important.

 Recent attempts at miRNA expression profiling may show the way toward more accurate classifications of human cancers than mRNA expression profiling [8]. Furthermore, the recent large-scale multidimensional analysis of molecular characteristics, TCGA, which includes expression profiles of miRNA along with DNA copy number, gene expression and DNA methylation, has revealed frequent genetic alterations in three critical core pathways [9]. In fact, Kim et al. identified five clusters of glioblastoma based on miRNA expression profiles, which appeared to predict clinical outcomes more precisely than mRNA profiles [24]. Expression profiles using miRNA are useful for subclassification of glioblastoma and could identify novel therapeutic targets.

## 8. Future Directions

 Since the introduction of temozolomide (TMZ) as standard chemotherapy, concomitant TMZ and radiotherapy have improved both progression-free survival and overall survival in patients with GBM [40]. However, the clinical prognosis of patients with GBM remains poor, with a median overall survival of only 14.6 months [40]. The cancer stem cell (CSC) hypothesis, that tumors are driven by a subpopulation of tumor cells with stem cell-like properties, may provide new insights into the radio- and chemo-resistance of GBM. Based on this hypothesis, some therapies in which shrink tumors show reduced diameter might not be associated with improved rates of cure if CSCs fail to be eliminated, and the therapies targeting CSCs should prove clinically more effective [41]. Elucidating the molecular biology of CSCs would thus be crucial.

 Recently, some miRNAs have been reported to contribute to CSC properties and cancer heterogeneity. In addition to miR-128 described above, miR-124, -137 [42], -34a [43, 44], and -326 [45] have been reported to play roles in the maintenance of CSC properties. The identification of miRNAs thus adds another layer of gene expression associated with gliomagenesis and maintenance of CSC properties via regulation of targeted signaling pathways. The regulation of aberrant miRNAs could affect not only conventional radio- and chemo-therapy, but also the sensitivity to molecular target therapy. Further study of miRNAs function in CSC may lead to novel treatment strategies for GBM patients. Consideration of the therapeutic application of miRNAs suggests two putative options: inhibition of oncogenic miRNAs and replacement of tumor-suppressor miRNAs. Development of the ideal delivery systems to target tissue remains under investigation. However, miRNA-based therapy could be more practical than gene therapy, since miRNA is a substantially small molecule.

 In conclusion, miRNA appears to offer a crucial biomarker not only as a diagnostic marker, but also as a target for molecular therapies. The miRNA approach to therapy could thus achieve modest changes for multiple genes and may prove more effective than current molecular targeted therapies to target single gene within an oncogenic signaling pathway. Molecular-targeted therapy based on miRNA expression in CSCs has the potential to allow more personalized and effective treatment strategies in the not-too-distant future.

## Figures and Tables

**Table 1:** Dysregulated miRNAs in glioblastoma.

Authors	Year	Reference	Methods	Number of miRNA	Comparison	Upregulation	Downregulation
Guan et al.						miR-15b	miR-105
						**miR-21 **	**miR-128-2 **
						miR-135(b)	miR-184
						miR-196a	miR-302b
	2010	[19]	TaqMan array	365	GBM versus AA	miR-196b	miR-302d
						miR-363	miR-367
							miR-383
							miR-504
							miR-517c
							miR-601

Ciafrè et al.	2005	[13]	Microarray	245	GBM versus NB	miR-9	**miR-128-1 **
						**miR-10b**	miR-181a
						**miR-21**	miR-181b
						miR-25	miR-181c
						miR-123	
						miR-125b-1	
						miR-125b-2	
						miR-130a	
						miR-221	

Chan et al.	2005	[12]	Microarray	180	GBM versus NB	**miR-21**	miR-188
						miR-135(b)	miR-198
						miR-138	miR-202
						miR-291-5^′^	
						miR-347	

Godlewski et al.	2008	[15]	Microarray	161	GBM versus ANB	**miR-10b**	miR-124a
						**miR-21**	**miR-128-1 **
						miR-26	**miR-128-2 **
						miR-383	miR-137
						miR-451	miR-139
						miR-486	miR-190
						miR-516-35p	miR-218
						miR-519d	miR-299
							miR-323
							miR-483
							miR-511-1

Sasayama et al.	2009	[16]	Microarray	127 or 188	GBM versus ANB	**miR-10b **	miR-134
						**miR-21 **	miR-302c
						miR-92b	miR-329
						miR-106b	miR-369-3p
						miR-183	miR-379

Malzkorn et al.	2010	[20]	TaqMan	157	GBM versus DA	miR-9	miR-184
						miR-15a	miR-328
						miR-16	
						miR-17	
						miR-19a	
						miR-20a	
						**miR-21 **	
						miR-25	
						miR-28	
						miR-130b	
						miR-140	
						miR-210	

Rao et al.	2010	[22]	LNA array	756	GBM versus AA	miR-16	**miR-128-1 **
						**miR-21**	**miR-128-2 **
						miR-22	miR-219-2-3p
						miR-24	
						miR-34a	
						miR-126	
						miR-142-5p	
						miR-143	
						miR-146b-5p	
						miR-155	
						miR-193a-3p	
						miR-199a/b-3p	
						miR-335	
						miR-376c	
						miR-381	
						miR-451	
						miR-509-3-5p	
						miR-513a-5p	
						miR-552	
						miR-886-3p/5p	

GBM: glioblastoma, AA: anaplastic astrocytoma, NB: normal brain, ANB: adjacent normal brain, and DA: diffuse astrocytoma. Bold: common dysregulated miRNA (more than 3 reports).
